# Comparative Analysis of Microbial Community Structure and Functional Traits of Baijiu Daqu Across Diverse Geographical Regions in China

**DOI:** 10.3390/foods15122182

**Published:** 2026-06-17

**Authors:** Feirong Bai, Chengshan Cai, Tianci Zhang, Ling Xu, Yunzhen Liu, Rui Liu, Ziying Ma, Minghui Jiang, Jiaqi Gao, Jingjing Zhang, Xuejian Yu, Tengfei Tang, Juan Chen, Su Yao

**Affiliations:** 1 Key Laboratory of Precision Nutrition and Food Quality, Department of Nutrition and Health, China Agricultural University, Beijing 100083, China; baifeirong@china-cicc.org; 2 China Center of Industrial Culture Collection, China National Research Institute of Food & Fermentation Industries, Beijing 100015, China; caichengshan@china-cicc.org (C.C.); zhangtianci@china-cicc.org (T.Z.); liuyunzhen@china-cicc.cn (Y.L.); liurui@china-cicc.org (R.L.); maziying@china-cicc.org (Z.M.); gaojiaqi@china-cicc.org (J.G.); zhangjingjing@china-cicc.cn (J.Z.); yuxuejian@china-cicc.org (X.Y.); tangtf@china-cicc.cn (T.T.); 3 Shandong Bandaojing Co., Ltd., Zibo 256300, China; xuling@bandaojing.cn (L.X.); jiangminghui@bandaojing.cn (M.J.)

**Keywords:** fermentation starter, solid-state fermentation, metagenomic sequencing, untargeted metabolomics analysis, metabolic pathways

## Abstract

*Daqu* is a key starter used in Baijiu production, and its microbial composition and associated metabolic functions play critical roles in fermentation performance and flavor development. This work aimed to reveal how *Daqu*-making temperature regulates microbial community divergence and subsequent metabolite formation via multi-omics analysis so as to provide theoretical guidance for *Daqu* quality control. In this study, physicochemical analysis, metagenomic sequencing, and metabolomic profiling were combined to investigate the microbial community structure, functional differentiation, and metabolite characteristics of nine *Daqu* samples collected from six major Baijiu-producing regions in China. The temperature during *Daqu* preparation was found to be a primary factor driving microbial community assembly and functional specialization. Medium-temperature *Daqu* exhibited higher saccharifying activity (up to 867 U) and greater microbial diversity with the enrichment of amino acid metabolism-related pathways, indicating enhanced protein degradation and amino acid utilization for the formation of flavor precursors. In contrast, high-temperature *Daqu* showed stronger capacities for carbohydrate degradation and conversion, particularly in starch and sucrose metabolism, which were closely associated with the enrichment of thermotolerant fungi and bacteria. LEfSe analysis identified 47 distinct microbial biomarkers (LDA score > 3.0), which could differentiate between medium- and high-temperature *Daqu*. Redundancy analysis indicated that environmental factors (moisture and acidity) together with functional properties (fermentation, esterification, liquefaction, and saccharification) act as key drivers of microbial functional patterns. Metabolomic analysis further revealed that medium-temperature *Daqu* had higher abundances of esters and fatty acids, whereas high-temperature *Daqu* had higher proportions of alcohols and ketones. Taken together, these results provide a multi-omics perspective on temperature-driven microbial functional differentiation in *Daqu* and offer a scientific basis for quality-oriented regulation and process optimization in Baijiu production.

## 1. Introduction

Chinese Baijiu, a distilled alcoholic beverage and one among the six world-famous distilled spirits, is traditionally produced from grains (such as sorghum, wheat, rice, and corn) through solid-state fermentation, and owes its unique brewing process to its intricate and diverse microbial ecosystem [1]. *Daqu*, a brick-shaped fermentation starter primarily composed of wheat, barley, and peas [2], which serves as the saccharifying, fermenting, and flavor-producing agent in Baijiu production, exerts a direct impact on the yield, quality, and flavor profile of the base liquor through its microecological structure and physicochemical properties [3]. Based on the peak temperature during *Daqu*-making, *Daqu* is generally classified into medium-temperature *Daqu* (45–50 °C), medium–high-temperature *Daqu* (50–60 °C), and high-temperature *Daqu* (>60 °C). *Daqu* is produced by shaping cereal substrates into bricks followed by spontaneous solid-state fermentation, during which microbial metabolic heat serves as the primary heat source. The peak temperature is controlled by adjusting the stacking density and turning frequency of the bricks. It harbors a complex microbial community composed of bacteria, fungi, and *Actinomycetes*, which produces abundant enzyme systems (e.g., amylases, esterases, and proteases) and flavor precursors through metabolic activities, collectively driving the Baijiu fermentation process [4,5].

In recent years, the advancement of high-throughput metagenomics has greatly expanded our understanding of the composition of the *Daqu* microbial community, revealing its remarkably high species diversity [6,7]. The emergence of untargeted metabolomics has provided a powerful tool for investigating the products of the *Daqu* microbial community. This technology enables a comprehensive analysis of differences in metabolite composition, thereby establishing a crucial bridge linking microbial communities to the functional quality of *Daqu* [8]. Recent studies have generally focused on specific types of *Daqu* or those from specific production areas. Examples of such studies include one analyzing the dynamic variation in thermophilic *Actinomycetes* in high-temperature *Daqu* for sesame-flavored Baijiu [4] and another study conducting a comparative analysis of fungal communities between medium-temperature and high-temperature *Daqu* from the same region [5]. However, systematic comparative studies spanning multiple geographical production areas and simultaneously covering the two major types (medium- and high-temperature) remain relatively limited. The lack of such systematic research hinders our ability to macroscopically understand the correlations among geographical environments, production techniques, and microecological functions of *Daqu* [9].

In this study, nine *Daqu* samples (six medium-temperature *Daqu* and three high-temperature *Daqu*) were collected from multiple core Baijiu-producing regions: Luzhou, Deyang, and Yibin in Sichuan Province; Zunyi in Guizhou Province; Jingzhou in Hubei Province; Lianyungang in Jiangsu Province; Zibo in Shandong Province; and Sanmenxia in Henan Province. By integrating techniques, such as physicochemical index determination, metagenomic sequencing, and untargeted metabolomics, we analyzed the microbial community structure, predicted metabolic functions, screened differential microorganisms via LEfSe analysis, performed differential analysis of metabolites, and conducted Spearman’s correlation analysis to reveal potential associations between microorganisms and flavor metabolites. This study achieved the following: (1) systematically clarified the patterns in the variation in physicochemical properties among *Daqu* samples from different production areas and of different types; (2) elucidated the characteristics of community structure and functional genes at the metagenomic level, as well as the variation patterns of metabolite composition; and (3) explored the intrinsic correlations within the “microbial community–functional genes–physicochemical properties–metabolites” axis. This study aimed to provide a theoretical basis for deciphering the microbial community characteristics and metabolic pathways of different types of *Daqu* as well as for the exploitation of microbial resources from *Daqu* across various production regions.

## 2. Materials and Methods

### 2.1. Sample Collection

**Figure 1:**
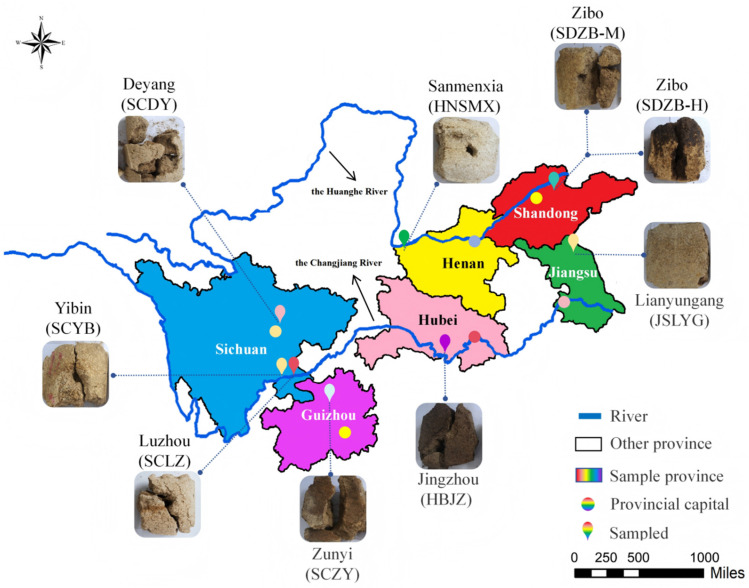
Schematic diagram of *Daqu* sampling.

In November 2023, we collected samples of a total of nine categories of *Daqu*, comprising six categories of medium-temperature *Daqu* from renowned Chinese liquor-producing regions, including Luzhou, Deyang, and Yibin in Sichuan Province, Lianyungang in Jiangsu Province, Sanmenxia in Henan Province, and Zibo in Shandong Province, and three categories of high-temperature *Daqu* from Zibo in Shandong Province, Jingzhou in Hubei Province, and Zunyi in Guizhou Province. The geographical distribution of the samples is shown in Figure 1. Samples were obtained from different spatial locations within the fermentation room: in the vertical dimension, samples were collected from the upper, middle, and lower layers of the *Daqu* stack; in the horizontal dimension of each layer, one *Daqu* brick was sampled from locations near the door, near the window, and at the center of the room, yielding a total of nine bricks per *Daqu* category as primary samples. Each brick was thoroughly pulverized, and nine pulverized samples from the same category were thoroughly homogenized to obtain a representative mixed sample for each *Daqu* type; these were subsequently stored at low temperatures for further analysis. The high-temperature *Daqu* samples collected in this study are primarily used for the production of sauce-aroma baijiu, which typically has an alcohol content of 53 vol% (commonly ranging from 50 to 55 vol%). The medium-temperature *Daqu* samples are primarily used for the production of strong-aroma baijiu, which typically has an alcohol content of 52 vol% (commonly ranging from 42 to 55 vol%).

### 2.2. Determination of Physicochemical Properties and Enzyme Activities

The moisture content, acidity, liquefying power, saccharifying power, fermenting power, and esterifying power were determined using standard methods according to the Chinese national light industry standard QB/T 4257-2011 [10]. Acidity was measured via acid–base neutralization potentiometric titration, expressed as millimoles of 0.1 mol/L sodium hydroxide standard solution consumed per 10 g of dry *Daqu*. Liquefying power was defined as the grams of starch liquefied by 1 g of dry *Daqu* at 35 °C and pH 4.6 within 1 h, which reflects the capacity of *Daqu* to hydrolyze starch into short-chain or small-molecule sugars (unit: g/g·h). Saccharifying power was defined as the milligrams of glucose produced by 1 g of dry *Daqu* from soluble starch conversion at 35 °C and pH 4.6 within 1 h, representing the saccharifying amylase activity involved in the hydrolysis of starch to monosaccharides (unit: mg/g·h). Fermenting power was defined as the grams of carbon dioxide produced by 0.5 g of *Daqu* from fermentable carbohydrates at 30 °C within 72 h, reflecting the ability of *Daqu* microorganisms to ferment sugars into alcohols, carbon dioxide, and other metabolites (unit: g/0.5 g·72 h). Esterifying power was defined as the milligrams of ethyl caproate synthesized by 50 g of *Daqu* within 7 d via catalyzing the reaction between caproic acid and ethanol at 35 °C, indicating the esterase activity of *Daqu* in the synthesis of esters from organic acids and alcohols (unit: mg/50 g·7 d).

### 2.3. Metagenomic DNA Extraction and Sequencing of Samples

A 10–15 g aliquot of each sample was taken and DNA extraction was performed using the Qiagen Soil DNA Kit (Qiagen, Hilden, Germany). Purity and integrity of the extracted DNA were evaluated using a BioDrop microvolume spectrophotometer (BioDrop, Cambridge, UK) and agarose gel electrophoresis, respectively. Qualified DNA samples were subjected to paired-end 150 bp (PE150) sequencing on the MGISEQ-2000 platform (MGI Tech, Shenzhen, China) by BGI-Shenzhen Co., Ltd. (Shenzhen, China).

### 2.4. Analysis of Microbial Community Structure and Function

Raw sequencing reads were processed and subjected to online data analysis using the BGI Dr. Tom Multi-omics Database Mining System (http://report.bgi.com [accessed on 15 October 2025]). The detailed procedures were as follows: SOAPnuke (v. 2.2.1) [11] was employed for filtering and quality control of the raw data. Reads containing 0.1% ambiguous bases, sequencing adapter sequences, or over 50% low-quality bases were discarded. Subsequently, Bowtie2 (v. 2.4.4) [12] was used to align the remaining reads against the host genome sequence, and the matched reads were removed to generate Clean Data for subsequent bioinformatics analysis. High-quality Clean Data were de novo assembled using MEGAHIT (v. 1.2.9) [13], with assembled contigs shorter than 200 bp filtered out. Ab initio gene prediction was performed using MetaGeneMark (v. 3.38) [14], followed by redundancy reduction of the predicted genes with CD-HIT (v. 4.8.1) [15]. Salmon (v. 1.6.0) [16] was applied to calculate the relative abundance table of each gene. Kraken2 (v. 2.1.2) [17] was used to align the Clean Data against the local National Center for Biotechnology Information (NCBI) Nucleotide (NT) database (Nt(202011)) for counting the sequence reads assigned to each taxon. Bracken2 (v. 2.6.1) [18] was then utilized to estimate the actual taxonomic abundance of each sample, thus completing species annotation. The BLASTP function of Diamond (v. 0.8.24) [19] was employed to align the non-redundant genes against multiple functional databases including eggNOG, Kyoto Encyclopedia of Genes and Genomes (KEGG), BacMet, Comprehensive Antibiotic Resistance Database (CARD), Clusters of Orthologous Groups (COG), Carbohydrate-Active Enzymes (CAZy), and Swiss-Prot, thereby achieving gene functional annotation. Based on the gene abundance table, species abundance table, and functional abundance table, visualization of gene, species, and functional distributions was conducted. Furthermore, Linear Discriminant Analysis Effect Size (LEfSe) analysis and KEGG pathway enrichment analysis were performed to identify the differences in species composition and functional composition among samples.

### 2.5. Untargeted Metabolomics Analysis

Headspace solid-phase microextraction (HS-SPME) coupled with gas chromatography-mass spectrometry (GC-MS) was employed for the qualitative and relative quantitative analysis of non-volatile metabolites in *Daqu* samples [20,21,22]. Specifically, a 7890B gas chromatograph (Agilent Technologies, Santa Clara, CA, USA) equipped with a 5977A mass spectrometer (Agilent Technologies, Santa Clara, CA, USA) and a DB-WAX capillary column (60 m × 0.25 mm × 0.25 μm; Agilent J&W Scientific, Folsom, CA, USA) was used. The instrumental setup consisted of an Agilent 7890 gas chromatograph and an Agilent 5975 mass spectrometer, equipped with an HP-5MS Ultra Inert capillary column (30 m × 0.25 mm × 0.25 μm, catalog No. 19091S-433UI). For metabolite extraction procedure, precisely 1 g of minced solid *Daqu* sample was taken in a sample vial, and 20 μL of internal standard solution (25 μg/mL deuterated styrene, CAS: 19361-62-7, dissolved in methanol) was added. Then, HS-SPME treatment was performed under the following conditions: equilibration at 60 °C with shaking at 250 rpm for 15 min, followed by headspace extraction at 60 °C for 30 min using an SPME fiber. After extraction, the fiber was inserted into the GC inlet for desorption at 260 °C for 5 min.

Chromatography and mass spectrometry had the following operating parameters. The inlet was operated in split mode with a split ratio of 25:1. High-purity helium (99.999%) was used as the carrier gas at a constant flow rate of 1.0 mL/min. The column temperature program was configured as follows: initial temperature maintained at 50 °C, ramped up to 230 °C at 7 °C/min and held for 3 min, then further increased to 320 °C at 40 °C/min and held for 5 min [23]. The mass spectrometry conditions were as follows: ion source temperature at 240 °C, quadrupole temperature at 160 °C, ionization energy at 70 eV, and a mass scan range of *m*/*z* 50–500.

Data processing and metabolite identification were performed through the following steps. First, the raw data were converted into the .abf format using AnalysisBaseFileConverter 2.0 software. Preprocessing procedures, including peak detection, peak alignment, and peak integration, were performed using MSDIAL 4.60 software [24,25]. Finally, qualitative analysis of the metabolites was performed by matching the obtained mass spectra and retention time indices against the NIST 2020 database [26].

### 2.6. Data Processing and Analysis

Based on the gene abundance table, species abundance table, and functional abundance table, LEfSe analysis and KEGG pathway enrichment analysis were performed on the BGI Dr. Tom online platform to identify inter-sample differences in species and functional composition. All statistical significance analyses were performed using SPSS 26.0 software. All statistical charts and visualization figures were generated with the online analytical platforms: PCA was finished on Novogene Magic-plus platform (https://magic-plus.novogene.com/ [accessed on 10 October 2025]), RDA was calculated via OmicStudio (https://www.omicstudio.cn/ [accessed on 21 October 2025]), and correlation network plots based on Spearman correlation coefficients were constructed using Gephi (v.0.9.2).

## 3. Results

### 3.1. Physicochemical Indices of Daqu

**Figure 2:**
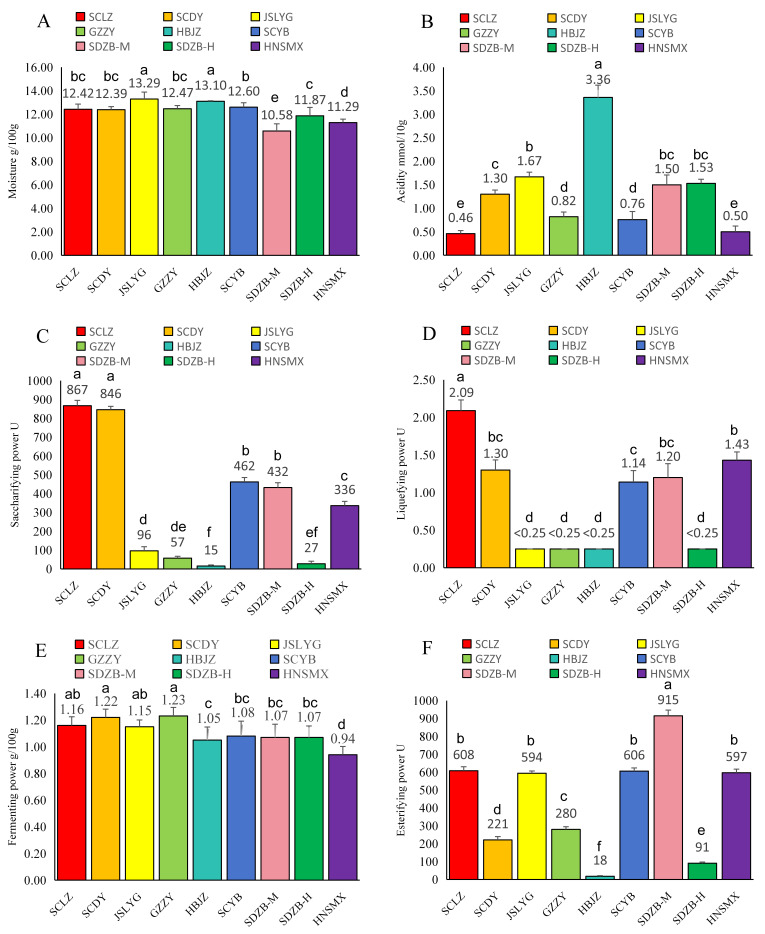
Determination of physicochemical indexes. (**A**) Moisture content, (**B**) total acidity, (**C**) saccharifying power, (**D**) liquefying power, (**E**) fermenting power, and (**F**) esterifying power of different *Daqu* samples. Different lowercase letters above the bars indicate statistically significant differences between samples (*p* < 0.05); samples sharing the same letter are not significantly different (*p* > 0.05). Sample abbreviations: SCLZ (Sichuan Luzhou), SCDY (Sichuan Deyang), JSLYG (Jiangsu Lianyungang), GZZY (Guizhou Zunyi), HBJZ (Hubei Jingzhou), SCYB (Sichuan Yibin), SDZB-M (Shandong Zibo, medium-temperature), SDZB-H (Shandong Zibo, high-temperature), HNSMX (Henan Sanmenxia).

The physicochemical indices, including moisture content, acidity, saccharifying power, liquefying power, fermenting power, and esterifying power, of the *Daqu* samples are presented in Figure 2. Medium-temperature *Daqu* generally exhibited excellent saccharification performance, with the SCLZ showing the highest saccharifying power of 867 U followed by SCDY with 846 U. Notably, the saccharifying power of high-temperature *Daqu* was generally low (15–57 U), which might be attributed to the inactivation of saccharifying enzymes caused by the high-temperature preparation process. In terms of esterification characteristics, SDZB-M *Daqu* showed an exceptionally high esterifying power (915 U), implying that it may harbor a unique microbial community composition. A correlation was observed between moisture content and enzyme activity—the JSLYG had the highest moisture content (13.29 g/100 g) and its saccharifying power (96 U) was the lowest among all the medium-temperature *Daqu* samples, which could be related to the adverse effects of excessive moisture on metabolic activities of microbes. Acidity indices indicated that high-temperature *Daqu* generally had higher acidity (0.82–3.36 mmol/10 g). Specifically, the HBJZ reached an acidity of 3.36 mmol/10 g, which was significantly higher than that of medium-temperature *Daqu* (0.46–1.67 mmol/10 g) (*p* < 0.01). Fermenting power indices were relatively stable (0.94–1.23 g/100 g), with the high-temperature *Daqu* GZZY exhibiting the highest value (1.23 g/100 g).

### 3.2. Composition and Differential Analysis of Microbial Community Structure

**Figure 3:**
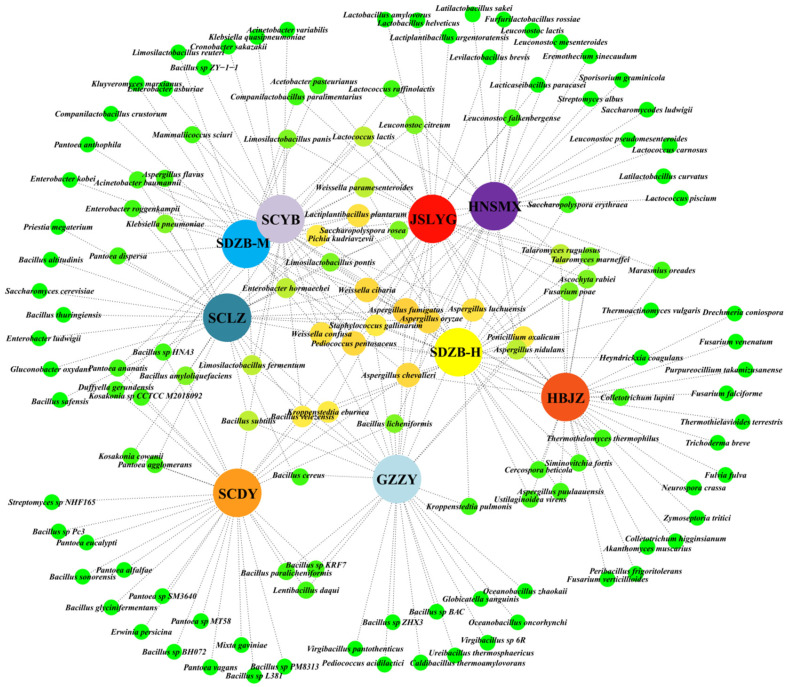
Metagenomic microbial community composition of different samples.

Metagenomic sequencing was performed to analyze the composition of the microbial community of nine *Daqu* samples (SDZB-H, SDZB-M, SCDY, GZZY, HBJZ, JSLYG, SCLZ, SCYB, and HNSMX). The species distribution across different samples is shown in Figure 3. At the species level, with a relative abundance greater than 1%, significant differences were observed in the dominant species among the samples. In sample SDZB-H, *Kroppenstedtia eburnea* had the highest relative abundance (46.43%), followed by *Aspergillus chevalieri* (23.74%). Other major species included *Staphylococcus gallinarum* (4.48%), *Aspergillus oryzae* (2.3%), *Pichia kudriavzevii* (1.45%), *Enterobacter hormaechei* (1.29%), and *Thermoactinomyces vulgaris* (1.1%). The microbial community of SDZB-M was dominated by *Pichia kudriavzevii* (14.81%) and *Limosilactobacillus pontis* (14.23%), with an additional 12 species, including *Kosakonia cowanii* (12.04%), *Weissella confusa* (8.44%), and *Pantoea agglomerans* (7.27%), each with a relative abundance greater than 1%. In SCDY, *Pantoea agglomerans* was the most abundant species (28.05%), followed by *Bacillus velezensis* (19.05%), and 15 other species, such as *Bacillus* sp. KRF7 (6.89%) and *Bacillus subtilis* (4.52%), also exhibited relative abundances exceeding 1%. The dominant species in GZZY were *Lentibacillus daqui* (53.8%) and *Kroppenstedtia eburnea* (30.86%), collectively accounting for over 80% of the microbial community, with *Bacillus licheniformis* (2.69%) and *Aspergillus chevalieri* (1.79%) being the sub-dominant species. HBJZ was characterized by a fungal-dominated community structure, with *Aspergillus oryzae* (21.45%), *Aspergillus luchuensis* (12.81%), and *Aspergillus chevalieri* (7.48%) being the top three dominant species, with 16 other species, including *Penicillium oxalicum* (6.3%) and *Aspergillus fumigatus* (5.17%), each with a relative abundance greater than 1%. In JSLYG, *Pichia kudriavzevii* had the highest relative abundance (31.36%), followed by *Kroppenstedtia eburnea* (9.44%) and *Lactobacillus helveticus* (7.98%), with a total of 16 species showing a relative abundance of >1%. SCLZ was dominated by *Limosilactobacillus fermentum* (20.18%) and *Pichia kudriavzevii* (14.96%), along with 14 other species, including *Weissella confusa* (8.81%) and *Bacillus velezensis* (6.73%), each with a relative abundance > 1%. In SCYB, *Weissella confusa* was the most abundant species (17.85%), followed by *Weissella paramesenteroides* (7.28%) and *Klebsiella pneumoniae* (5.62%), with 19 species in total showing a relative abundance > 1%. The dominant species in HNSMX were *Latilactobacillus curvatus* (20.99%), *Saccharopolyspora rosea* (11.33%), and *Leuconostoc citreum* (10.38%), accompanied by nine other species, including *Weissella cibaria* (6.72%), each with a relative abundance > 1%.

### 3.3. LEfSe2 Analysis

**Figure 4:**
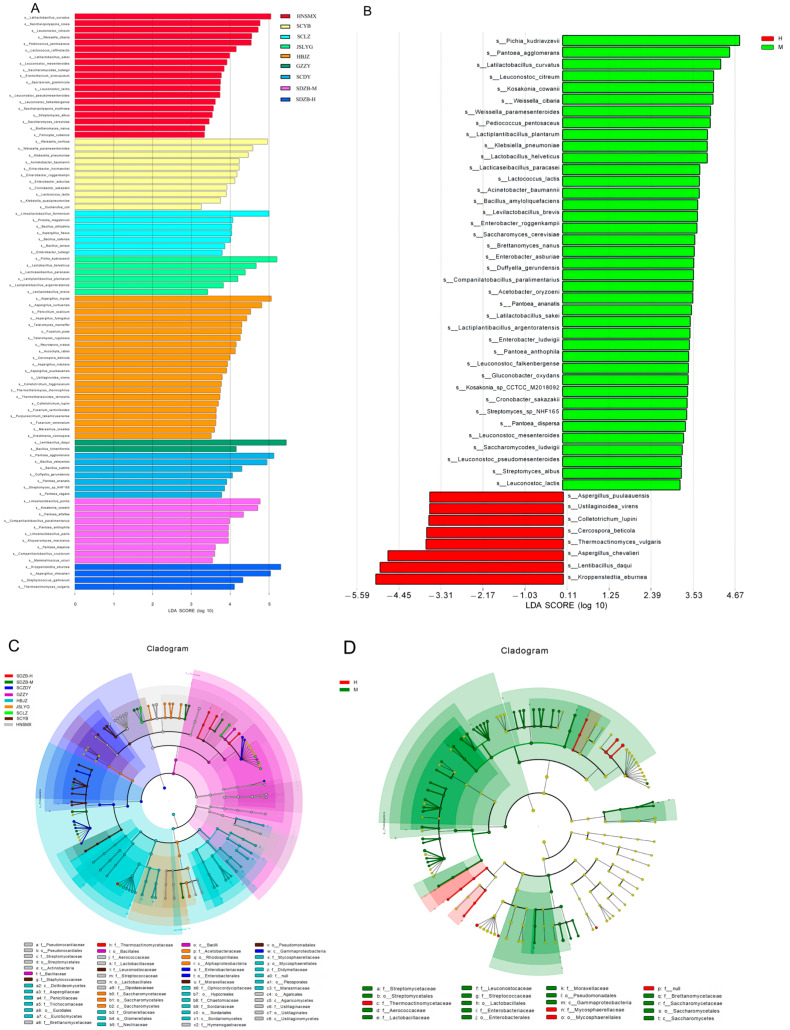
LEfSe analysis results. (**A**) LDA score plot for different *Daqu* samples; (**B**) LDA score plot for medium–high-temperature *Daqu*; (**C**) Cladogram exhibiting the evolutionary relationship for different *Daqu* samples; (**D**) Cladogram exhibiting the evolutionary relationship for medium–high-temperature *Daqu*.

LEfSe2 analysis showed that when the nine *Daqu* samples were analyzed as individual units, the specific microbial taxa varied significantly among different samples (Figure 4). The characteristic microbial taxa of HNSMX included 19 groups, such as *Latilactobacillus curvatus*, *Saccharopolyspora rosea*, and *Leuconostoc citreum*. SCYB was characterized by 11 taxa including *Weissella confusa*, *Weissella paramesenteroides*, and *Klebsiella pneumonia*. The key differential microorganisms of SCLZ comprised seven taxa including *Limosilactobacillus fermentum* and *Priestia megaterium*. The characteristic microorganisms of JSLYG included six taxa such as *Pichia kudriavzevii* and *Lactobacillus helveticus*. HBJZ was characterized by 22 taxa including *Aspergillus oryzae* and *Aspergillus luchuensis*. The characteristic microorganisms of GZZY were *Lentibacillus daqui* and *Bacillus licheniformis*. SCDY was characterized by seven taxa such as *Pantoea agglomerans* and *Bacillus velezensis*. The characteristic microorganisms of SDZB-M comprised 10 taxa including *Limosilactobacillus pontis* and *Kosakonia cowanii*. SDZB-H was characterized by four taxa such as *Kroppenstedtia eburnea* and *Aspergillus chevalieri*. After grouping the nine samples into medium- and high-temperature groups, LEfSe2 analysis revealed significant differences in the microbial community structure between the two groups. The dominant differential microorganisms of medium-temperature *Daqu* were mainly yeasts and bacteria: the yeast group included four taxa such as *Pichia kudriavzevii* and *Saccharomyces cerevisiae*; and the bacterial group covered *Enterobacter* spp. (*E. roggenkampii*, *E. asburiae*, *E. ludwigii*), *Pantoea* spp. (four species including *Pantoea agglomerans*), *Lactobacillus*-related genera (eight taxa including *Latilactobacillus curvatus*), *Leuconostoc* spp. (five species including *Leuconostoc citreum*), as well as 12 other genera such as *Kosakonia*, *Weissella*, and *Pediococcus*. The characteristic taxa of high-temperature *Daqu* were dominated by fungi and thermotolerant bacteria, including *Aspergillus* spp. (*Aspergillus puulaauensis* and *A. chevalieri*), *Ustilaginoidea virens*, *Colletotrichum lupini*, *Thermoactinomyces vulgaris*, *Cercospora beticola*, *Lentibacillus daqui*, and *Kroppenstedtia eburnea*.

### 3.4. Redundancy Analysis (RDA) of Correlations Between Dominant Microbial Communities and Physicochemical Properties

**Figure 5:**
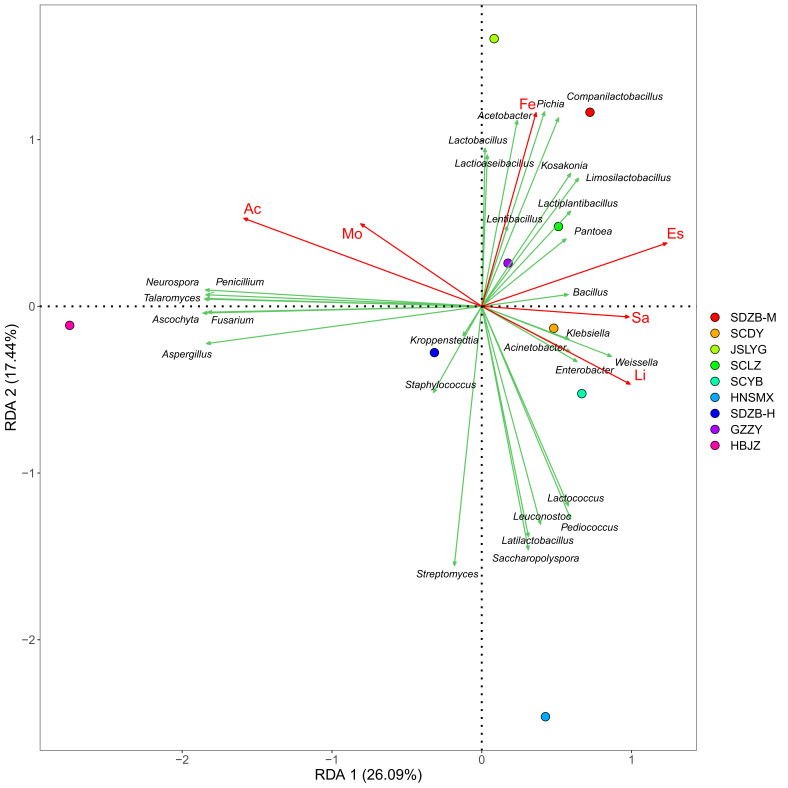
Redundancy analysis of the correlation between dominant communities and physicochemical properties. The six environmental indices included acidity (Ac), moisture (Mo), saccharifying power (Sa), liquefying power (Li), fermenting capacity (Fe), and esterifying capacity (Es). Green arrows indicate microbial genera, while red arrows represent physicochemical indices.

RDA revealed significant correlations between microbial community structure and environmental and functional indices in *Daqu* samples (Figure 5). In the second quadrant, the arrows for both acidity (Ac) and moisture (Mo) pointed to the upper left, which is highly consistent with the distribution of fungi such as *Neurospora*, *Penicillium* and *Talaromyces*. This indicated a strong positive correlation between these fungal taxa and environments with high moisture content and acidity levels, suggesting that they may coexist in humid and acidic *Daqu* microenvironments and potentially participate in acid production. The arrows for fermenting power (Fe) and esterifying power (Es) pointed to the first quadrant, showing close associations with multiple microbial taxa including *Pichia*, *Acetobacter*, various lactic acid bacteria (LAB) (e.g., *Lactobacillus* and *Lacticaseibacillus*), and *Bacillus*. Among these, the arrow for *Pichia* was long and co-directional with that of Fe, implying that *Pichia* may play a key role in the fermentation process. The angle between the Es and *Bacillus* was the smallest, and SCLZ and GZZY were located in close proximity to them. These findings further demonstrate the strong correlation between the genus *Bacillus* and esterification function as well as the active metabolic performance of this genus in these samples. In addition, the arrows for liquefying power (Li) and saccharifying power (Sa) showed similar directions, indicating considerable overlap in the microbial taxa that influence these processes. The arrow for *Enterobacter* was highly co-directional and comparable in length to that of Li, with SCYB and SCDY distributed nearby. This suggests a strong positive correlation between *Enterobacter* spp. and liquefaction. Moreover, *Weissella* was the closest to both Sa and Li and showed a strong association with SCDY, indicating that *Weissella* might play an important role in saccharification. Other taxa, such as *Klebsiella* and *Lactococcus,* were also distributed in this region, forming a functional microbial consortium responsible for liquefaction and saccharification. In the third quadrant, *Ascochyta* and *Fusarium* were highly codirectional with HBJZ, whereas *Kroppenstedtia* and *Staphylococcus* were closely located and codirectional with SDZB-H. The microbial community in this quadrant negatively correlated with the main functional indices in the second quadrant, which may represent a distinct microecological type that differs from those with high fermentation and esterification capacities.

### 3.5. Distribution and Differences in Metabolic Pathways of Different Daqu Samples

**Figure 6:**
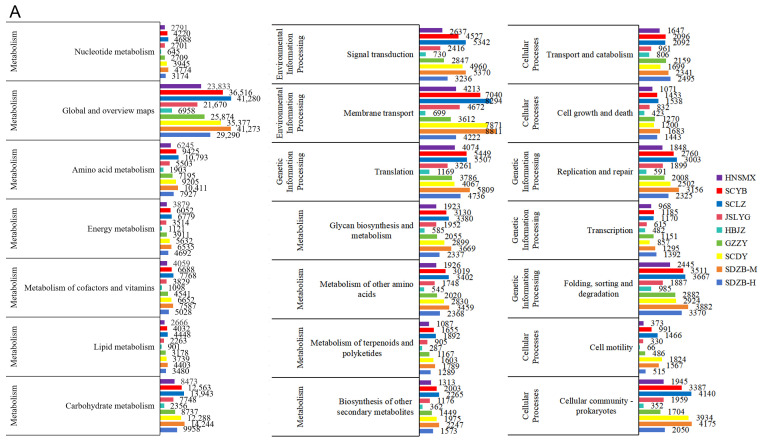

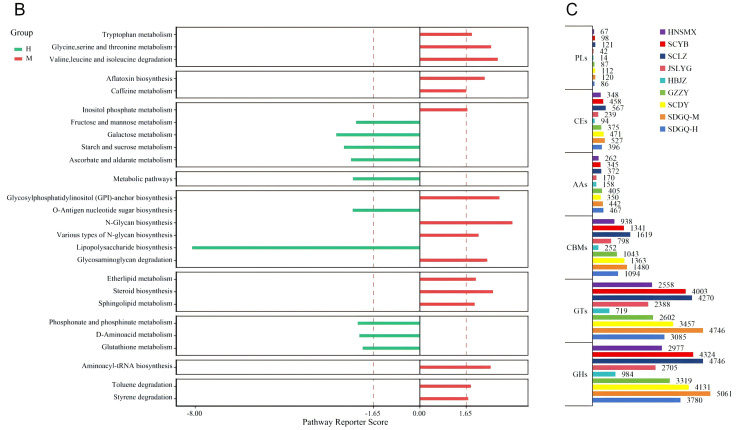
Distribution and differences in metabolic pathways among different *Daqu* samples. (**A**) KEGG pathway annotation plot of the nine *Daqu* samples. (**B**) KEGG pathway enrichment plot of medium−high−temperature *Daqu* samples. (**C**) Annotation plot based on the CAZy database for carbohydrate-active enzymes, including six enzyme families: GHs (Glycoside Hydrolases), GTs (Glycosyltransferases), CBMs (Carbohydrate-Binding Modules), AAs (Auxiliary Activities), CEs (Carbohydrate Esterases), PLs (Polysaccharide Lyases).

Metagenomic data were annotated against the KEGG and CAZy databases to explore discrepancies in functional characteristics and metabolic pathways across different *Daqu* samples, and the results are presented in Figure 6. KEGG pathway annotation categorized the results into six level 1 pathways and 28 level 2 pathways, revealing distinct variations in the gene abundance of each pathway among the samples. In the KEGG Level 1 classification, metabolism-associated genes had the highest abundance in the microbial communities of all *Daqu* samples, accounting for 71.31%, 70.23%, 70.95%, 72.30%, 70.66%, 71.63%, 71.38%, 71.16%, and 71.03% of the total annotated genes in SDZB-H, SDZB-M, SCDY, GZZY, HBJZ, JSLYG, SCLZ, SCYB, and HNSMX, respectively.

We further analyzed the distribution of metabolism-related functional genes in the KEGG Level 2 classification. Consistent with previous reports [27], carbohydrate metabolism and amino acid metabolism were the most prominent biological functions, indicating that *Daqu* possesses robust potential for substrate degradation and biosynthesis of flavor compounds.

After grouping the samples into medium- and high-temperature *Daqu*, KEGG pathway enrichment analysis showed that three pathways—starch and sucrose metabolism, galactose metabolism, and fructose and mannose metabolism—were simultaneously enriched in high-temperature *Daqu*. This indicates that the microbial community in high-temperature *Daqu* has a strong capacity for the decomposition and transformation of major carbohydrates (e.g., starch and sucrose) as well as monosaccharides (e.g., galactose and fructose). Under high-temperature conditions, microorganisms must rapidly utilize carbohydrates to generate energy to sustain vital activities, and their metabolic products (e.g., small-molecule sugars and alcohols) may serve as precursors for flavor formation in *Daqu*.

In contrast, multiple amino acid-related pathways, including valine-leucine-isoleucine degradation, glycine-serine-threonine metabolism, tryptophan metabolism, and d-amino acid metabolism, were enriched in medium-temperature *Daqu*. This indicates that microorganisms in medium-temperature *Daqu* have a high capacity for protein degradation and amino acid utilization. A medium-temperature environment is more conducive to protease activity, enabling microorganisms to efficiently degrade proteins in raw materials and utilize amino acids as nitrogen sources for energy supply or to convert them into flavor compounds (e.g., aldehydes and ketones). This constitutes an important metabolic basis for the diversity of flavor substances in medium-temperature *Daqu*. These differences between medium- and high-temperature *Daqu* clearly illustrate the factors involved in the temperature-driven differentiation of metabolic functions in *Daqu*-making microorganisms.

Considering that carbohydrate metabolism has the highest abundance in the *Daqu* microbial community, and starch is the most abundant component in raw materials, it is crucial to investigate the differences in carbohydrate-utilization capacity among different *Daqu* samples based on the CAZy database. Among the six annotated functional categories, glycoside hydrolases (GHs) and glycosyltransferases (GTs) were dominant in all samples, which is consistent with previous studies [28]. GHs are a class of important enzymes that hydrolyze glycosidic bonds, including those involved in starch liquefaction, saccharification, and cellulose degradation, whereas GTs are mainly involved in carbohydrate biosynthesis, namely oligosaccharides and polysaccharides [1]. The abundances of GH families, ranked from highest to lowest, were as follows: SDZB-M (5061), SCLZ (4746), SCYB (4324), SCDY (4131), SDZB-H (3780), GZZY (3319), HNSMX (2977), JSLYG (2705), and HBJZ (984). For GT families, the abundance ranking was in the following order: SDZB-M (4746), SCLZ (4270), SCYB (4003), SCDY (3457), SDZB-H (3085), GZZY (2602), HNSMX (2558), JSLYG (2388), and HBJZ (719).

Overall, the transcripts per million (TPM) gene abundances of GHs and GTs in medium-temperature *Daqu* were higher than those in high-temperature *Daqu*. This provides an important rationale for the significantly higher dosage of high-temperature *Daqu* (50%) than that of medium-temperature *Daqu* (25%) in Baijiu brewing, as sufficient degradation of substrates is an indispensable prerequisite for subsequent ethanol fermentation [29].

### 3.6. PCA Analysis of Non-Volatile Metabolites

Headspace solid-phase microextraction (HS-SPME) coupled with gas chromatography-mass spectrometry (GC-MS) was employed for the qualitative and relative quantitative analysis of non-volatile metabolites in *Daqu* samples, with a total of 665 compounds identified across the nine samples. The major metabolite categories in *Daqu* included esters, alkanes (including branched-chain alkanes), alcohols, aldehydes and ketones, organic acids, pyrazines (including nitrogen heterocycles), furans, terpenes/terpene alcohols, and sulfur-containing compounds, and the relative abundances of these categories varied among different samples. Esters accounted for the highest proportion of all metabolite categories in the nine *Daqu* samples.

**Figure 7:**
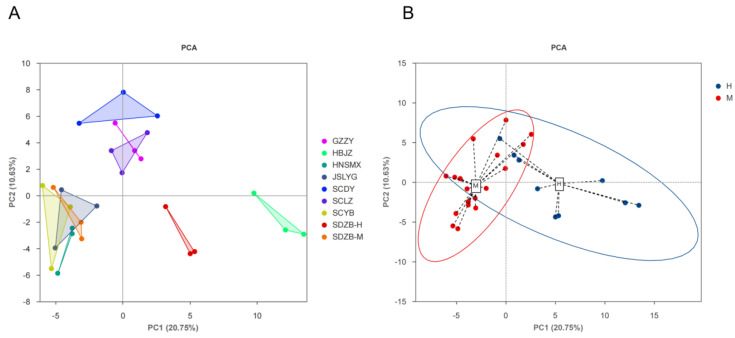
PCA results of non-volatile metabolites in different samples. (**A**) PCA plot of metabolites from the nine *Daqu* samples. (**B**) PCA plot of metabolites from medium−high−temperature *Daqu* samples.

A PCA plot was constructed based on the relative abundance of metabolites to distinguish between different samples and *Daqu* types grouped by temperature. The two principal components (PC1 and PC2) explained 31.38% of the total metabolic variation, revealing metabolite discrepancies among different *Daqu* samples (Figure 7A for individual samples and Figure 7B for temperature-based groups). Specifically, the PCA score plot showed that the nine *Daqu* samples were divided into four relatively distinct clusters in the PC1–PC2 dimension: SCDY, GZZY, and SCLZ formed one cluster, indicating high similarity in their metabolite composition patterns; JSLYG, SDZB-M, HNSMX, and SCYB were clustered together in another group, suggesting low intragroup variability in metabolite profiles; and SDZB-H and HBJZ were each distributed in separate, independent regions without clustering with other samples, which indicated that the metabolite profiles of these two samples were significantly unique compared with those of the other samples.

### 3.7. Differential Analysis of Daqu Metabolites

**Figure 8:**
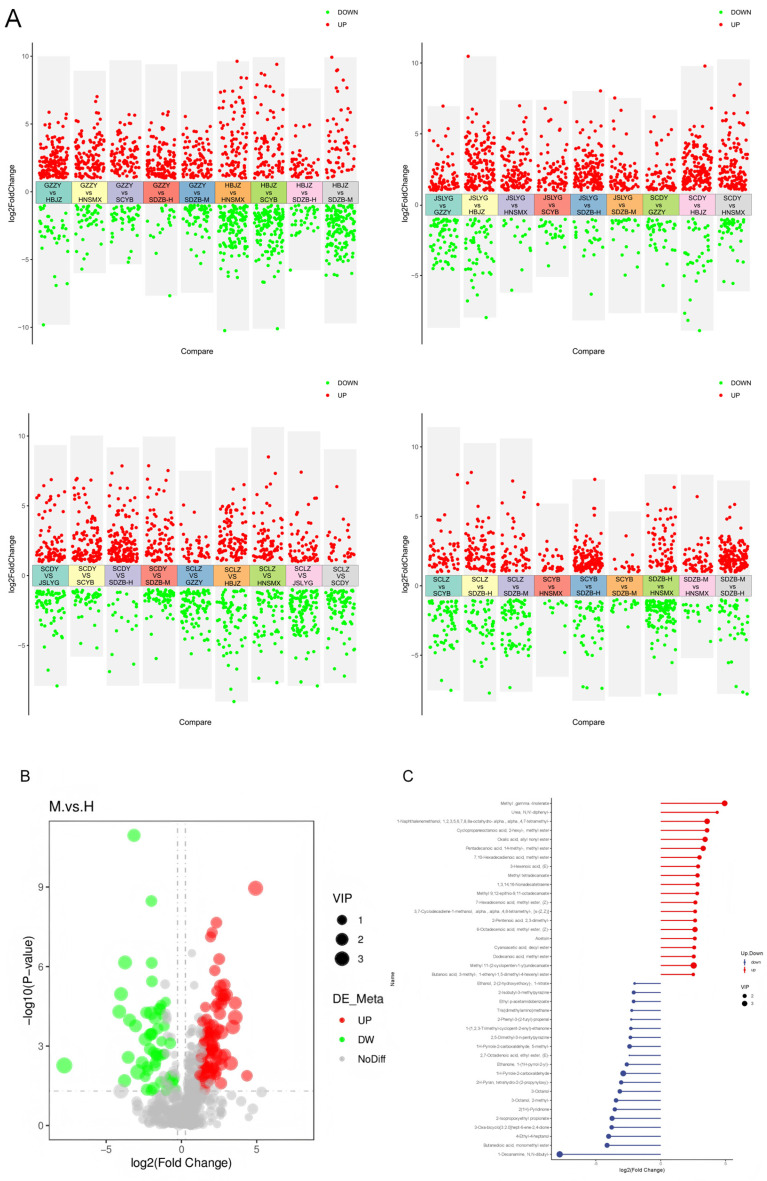
Differential analysis results of *Daqu* metabolites. (**A**) A volcano plot illustrating metabolite differences among the nine *Daqu* samples. (**B**) A volcano plot displaying metabolite differences among medium−high−temperature *Daqu* samples. (**C**) A match plot presenting metabolite differences among medium–high-temperature *Daqu* samples.

The results of differential metabolite screening are presented in Figure 8A, which shows considerable variation in the total number of significant differential metabolites across different sample pairs. The most significantly differentiated pairs were SCYB vs. HNSMX (125 metabolites) and SDZB-M vs. SDZB-H (115 metabolites), indicating the highest degree of divergence in metabolite composition between these groups. In contrast, pairs with fewer differential metabolites included SCYB vs. SDZB-M (30 metabolites) and SCDY vs. GZZY (62 metabolites), besides the aforementioned SCYB vs. HNSMX. This trend was consistent with the “clustering of partial samples” observed in the previous PCA plot, verifying the similarity in metabolic characteristics among these samples.

Analysis of the quantitative characteristics of intergroup differential metabolites revealed that, in most sample pairs, the number of significantly upregulated metabolites exceeded that of significantly downregulated metabolites. For instance, the SDZB-M vs. JSJYG pair showed 97 upregulated and 17 downregulated metabolites, whereas GZZY vs. SCYB showed 73 upregulated and 28 downregulated metabolites, suggesting more active metabolite expression in one sample of each pair. A few pairs exhibited a dominance of downregulated metabolites, such as SCLZ vs. SCDY (14 upregulated and 48 downregulated metabolites), implying the inhibition of certain metabolic pathways in one sample of the pair. Combined with the relevant conclusions from PCA, the quantitative results of differential metabolites in this table were consistent with the “four clustering regions” identified in the PCA plot—samples clustered together (e.g., SCDY, GZZY, and SCLZ) had fewer differential metabolites between pairs, whereas independently distributed samples (e.g., SDZB-H and HBJZ) showed a larger number of differential metabolites when paired with other samples, further validating the uniqueness of their metabolic characteristics.

After grouping the nine *Daqu* samples into high- and medium-temperature groups, 135 significantly differential metabolites were identified, accounting for approximately 20.3% of the total detected metabolites. Among these, 84 metabolites were upregulated and 51 were downregulated in medium-temperature *Daqu* (M). These data directly demonstrate a clear differentiation in the metabolite composition between *Daqu* of different temperature types, with a higher level of metabolic activation in medium-temperature *Daqu* (M). The upregulated metabolites were predominantly esters and fatty acids (e.g., methyl γ-linolenate, methyl octadecenoate), which was consistent with the flavor compound profile of medium-temperature *Daqu*. In contrast, the downregulated metabolites (shown in blue) were mostly alcohols and ketones, which correspond to the carbohydrate metabolism-related products of high-temperature *Daqu* (Figure 8C). The significant metabolic differences between medium-temperature *Daqu* (M) and high-temperature *Daqu* (H) directly reflect the temperature-driven differentiation of microbial metabolic functions during the production of *Daqu*.

### 3.8. Interactions Between Microorganisms and Metabolites

**Figure 9:**
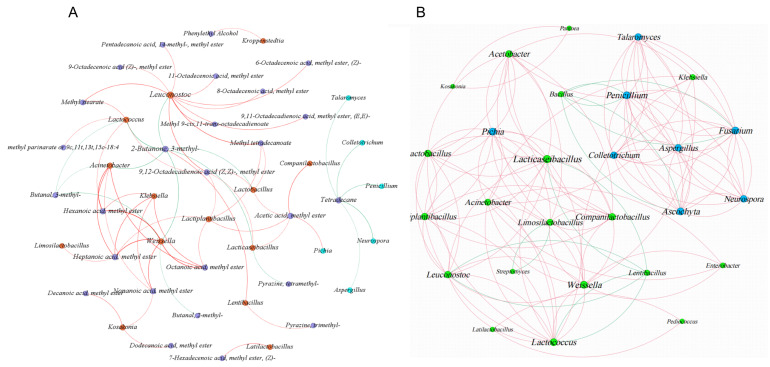
Spearman correlation analysis (*p* < 0.05, |r| > 0.7). Based on Spearman’s rank correlation analysis, an association network between metabolites and microorganisms was constructed (**A**), and the interaction relationships among microorganisms were also visualized via the network diagram (**B**). Orange, blue, and purple circles represent bacteria, fungi, and metabolites, respectively. Red and green lines represent positive and negative interactions, respectively. Line thicknesses are proportional to the value of Spearman’s correlation.

A Spearman’s rank correlation network was constructed to elucidate the interrelationships among microorganisms as well as the associations between microbial communities and metabolites. Correlations with statistical significance (*p* < 0.05, |r| > 0.7) were screened, based on which two networks were established. One network depicted the relationship between microbial communities and flavor metabolic pathways, and consisted of 46 nodes and 62 edges (Figure 9A). The second network characterized microbial interactions, and contained 29 nodes and 129 edges (Figure 9B).

Acinetobacter correlated with six metabolites: 3-methyl-2-butanone, 3-methylbutanal, and four fatty acid methyl esters. Five fungal genera—*Aspergillus*, *Colletotrichum*, *Neurospora*, *Penicillium*, and *Talaromyces*—were exclusively correlated with tetradecane. Five bacterial genera, *Companilactobacillus*, *Kroppenstedtia*, *Latilactobacillus*, *Limosilactobacillus*, and *Pichia*, were each associated with one specific metabolite, methyl acetate, phenylethanol, methyl (Z)-7-hexadecenoate, methyl heptanoate, and methyl acetate, respectively. *Klebsiella* and *Lactobacillus* were correlated with four metabolites each. The former was exclusively associated with fatty acid methyl esters, whereas the latter was associated with metabolites including 3-methyl-2-butanone and methyl linoleate. *Kosakonia* was correlated with three metabolites, whereas *Lentibacillus* was associated with two metabolites, 3-methyl-2-butanone and trimethylpyrazine. *Lacticaseibacillus* and *Lactiplantibacillus* were correlated with five metabolites each, including methyl linoleate and methyl acetate. *Lactococcus* was associated with seven metabolites. *Leuconostoc* exhibited correlation with the highest number of metabolites (13 species, including multiple fatty acid methyl esters), followed by *Weissella*, which was correlated with eight metabolites, including 3-methyl-2-butanone and various fatty acid methyl esters.

In terms of microbial interactions, a comprehensive positive correlation was observed among LAB species, including *Lacticaseibacillus*, *Lactiplantibacillus*, *Lactobacillus*, *Leuconostoc*, *Lactococcus*, *Companilactobacillus*, and *Limosilactobacillus*. These LAB species also showed positive correlations with the yeast *Pichia* and the acetic acid bacterium *Acetobacter*, thereby forming a core functional microbial consortium centered on “LAB-Yeast-Acetic acid bacterium” for fermentation in *Daqu*. *Acinetobacter* was positively correlated with *Weissella*, *Klebsiella*, *Enterobacter*, and *Lactococcus*. Additionally, positive correlation pairs were identified between *Kosakonia* and *Pantoea* and between *Latilactobacillus* and *Pediococcus*, which reflected the synergistic distribution characteristics of non-LAB in *Daqu*. A fully positive correlation was detected among filamentous fungi, such as *Aspergillus*, *Ascochyta*, *Colletotrichum*, *Fusarium*, *Neurospora*, *Penicillium*, and *Talaromyces*, which together constituted a stable fungal community structure.

Only three groups of negative correlations were identified in *Daqu*, indicating antagonistic effects between a small number of species. The bacterium *Bacillus* showed negative correlations with the fungi *Ascochyta*, *Fusarium*, and *Penicillium*, suggesting that the growth of *Bacillus* may inhibit the reproduction of certain filamentous fungi, or that competition for nutrients and spatial niches exists between them in the *Daqu* microenvironment. *Lentibacillus* was negatively correlated with LAB species, including *Lacticaseibacillus*, *Lactococcus*, and *Leuconostoc*, indicating growth inhibition or ecological niche competition between *Lentibacillus* and LAB groups. The actinomycete *Streptomyces* was negatively correlated with the LAB species *Limosilactobacillus*, reflecting a mutually exclusive relationship between actinomycetes and LAB in the *Daqu* ecosystem.

### 3.9. Prediction of Daqu-Related Metabolic Pathways and Enzyme Abundances

**Figure 10:**
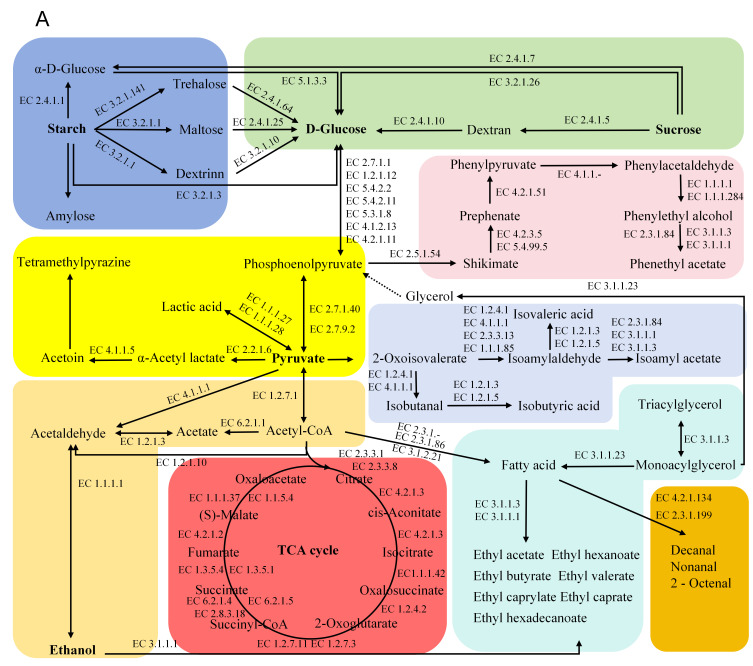

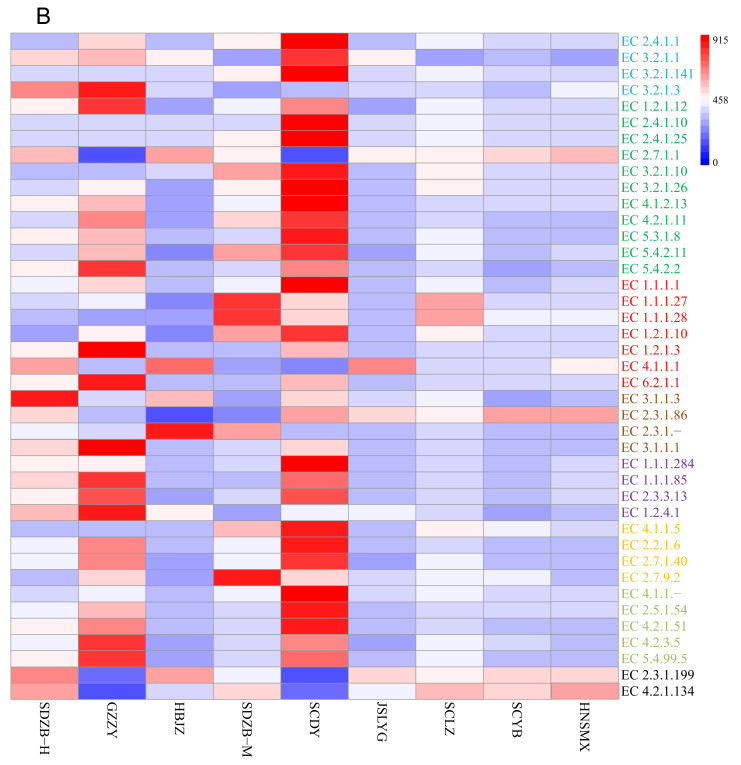
Metabolic pathway prediction (**A**) and the corresponding relationship between species and enzymes (**B**). The heatmap shows the relative abundance of key enzymes involved in substrate degradation and flavor compound synthesis. Enzyme names corresponding to EC numbers: EC 2.4.1.1 (glycogen synthase), EC 3.2.1.1 (α-amylase), EC 3.2.1.141 (maltose synthase), EC 3.2.1.3 (glucan 1,4-α-glucosidase), EC 1.2.1.12 (glyceraldehyde-3-phosphate dehydrogenase), EC 2.4.1.10 (maltooligosyltrehalose synthase), EC 2.4.1.25 (4-α-glucanotransferase), EC 2.7.1.1 (hexokinase), EC 3.2.1.10 (oligo-1,6-glucosidase), EC 3.2.1.26 (β-galactosidase), EC 4.1.2.13 (fructose-bisphosphate aldolase), EC 4.2.1.11 (phosphopyruvate hydratase), EC 5.3.1.8 (triose-phosphate isomerase), EC 5.4.2.11 (phosphoglucomutase), EC 5.4.2.2 (phosphoglycerate mutase), EC 1.1.1.1 (alcohol dehydrogenase), EC 1.1.1.27 (lactate dehydrogenase), EC 1.1.1.28 (D-lactate dehydrogenase), EC 1.2.1.10 (acetaldehyde dehydrogenase), EC 1.2.1.3 (aldehyde dehydrogenase), EC 4.1.1.1 (pyruvate decarboxylase), EC 6.2.1.1 (acetate-CoA ligase), EC 3.1.1.3 (triacylglycerol lipase), EC 1.1.1.284 (S-(hydroxymethyl)glutathione dehydrogenase), EC 1.1.1.85 (3-oxoacyl-[acyl-carrier-protein] reductase), EC 2.3.3.13 (2-ethylmalate synthase), EC 1.2.4.1 (pyruvate dehydrogenase), EC 4.1.1.5 (2-oxoglutarate decarboxylase), EC 2.2.1.6 (acetolactate synthase), EC 2.7.1.40 (pyruvate kinase), EC 2.7.9.2 (pyruvate, phosphate dikinase), EC 4.1.1.- (carboxy-lyase), EC 2.5.1.54 (3-deoxy-7-phosphoheptulonate synthase), EC 4.2.1.51 (prephenate dehydratase), EC 4.2.3.5 (chorismate synthase), EC 5.4.99.5 (chorismate mutase), EC 2.3.1.199 (acyltransferase), EC 4.2.1.134 (hydroxyacylglutathione hydrolase), EC 3.1.1.1 (carboxylesterase), EC 2.3.1.86 (fatty acyltransferase), EC 2.3.1.-(acyltransferase). EC numbers in different colors are involved in distinct metabolic processes: blue fonts for starch liquefaction, green fonts for saccharification and ester synthesis, red fonts for alcoholic fermentation, brown fonts for substrate hydrolysis and amino acid metabolism, purple fonts for higher alcohol biosynthesis, orange fonts for tetramethylpyrazine biosynthesis, light green fonts for phenylethyl alcohol biosynthesis, and black fonts for fatty acid biosynthesis.

To clarify the expression levels of enzyme-encoding genes associated with substrate degradation and characteristic flavor metabolism as well as the contributions of core microorganisms, we reconstructed a metabolic network encompassing nine metabolic pathways based on KEGG database annotations (Figure 10). A total of 41 related enzymes were screened and their abundances were visualized using a heatmap. The results of key enzyme abundances in each module indicated that the abundances of enzymes related to liquefying power; saccharifying power; fermenting power; and the synthesis of higher alcohols, tetramethylpyrazine, and phenylethanol in SCDY were higher than those in other samples. Among the remaining samples, the abundances of enzymes related to liquefying power, saccharifying power, fermenting power, and the synthesis of tetramethylpyrazine and phenylethanol in GZZY and SDZB-M were lower than those in SCDY; however, they were higher than those in the other samples. The abundances of enzymes related to synthesis of higher alcohols in GZZY and SDZB-H were lower than those in SCDY; however, they were higher than those in the other samples. SCDY, SDZB-H, and HBJZ exhibited relatively high abundances of enzymes related to esterification reactions, whereas SDZB-H and HBJZ showed high abundances of enzymes related to fatty acid synthesis. The enzyme abundances in the other samples were relatively low.

Metabolic pathway analysis revealed that starch was catalytically liquefied by enzymes such as glycogen synthase (EC 2.4.1.1) and maltose synthase (EC 3.2.1.141), yielding products including α-D-glucose and trehalose. Simultaneously, sucrose was converted to glucan under the action of sucrose phosphorylase (EC 2.4.1.5), which was subsequently degraded to glucose. The liquefied products were further subjected to saccharification catalyzed by enzymes such as α-glucan branching enzyme (EC 2.4.1.41) and oligo-1,6-glucosidase (EC 3.2.1.10), generating D-glucose, which served as a sufficient carbon source for downstream metabolic processes. Glucose was sequentially converted into phosphoenolpyruvate (PEP) and pyruvate through a series of enzymatic reactions and finally transformed into ethanol. During the esterification stage, acetyl-CoA or triglycerides underwent enzymatic reactions to produce fatty acids, which then reacted with ethanol to synthesize various lipids, such as ethyl acetate and ethyl caproate. Tetramethylpyrazine was enzymatically synthesized from pyruvate via the α-acetolactate–acetoin pathway. Phenylethanol was produced from PEP via the shikimate pathway and ultimately converted to ethyl phenylacetate. In addition, fatty acids were catalyzed by enzymes, including hydroxyacylglutathione hydrolase (EC 4.2.1.134), to generate decanal, nonanal, and other aldehydes. Using 2-oxoisovaleric acid as a precursor, higher alcohols were synthesized via two branched enzymatic pathways, producing isobutyric acid, isovaleric acid, isoamyl acetate, and related derivatives [30].

## 4. Discussion

Analysis of physicochemical indices revealed a significant divergence in key enzyme activities between medium- and high-temperature *Daqu*. Medium-temperature *Daqu* generally exhibited higher saccharifying power (up to 867 U) and esterifying power (up to 915 U), whereas the saccharifying power of high-temperature *Daqu* was significantly lower (15–57 U). This finding is consistent with the conclusions of Yi et al. regarding the temperature adaptability of high-temperature *Daqu*, which stated that high-temperature stress selectively preserves microorganisms associated with thermotolerant enzyme systems, thereby leading to a reduction in the activity of conventional saccharifying enzymes [23]. Notably, SDZB-M showed an exceptionally high esterifying power. Combined with the results of the LEfSe analysis, the characteristic microbial taxa of SDZB-M were dominated by *Pantoea* and *Limosilactobacillus*. Previous studies have confirmed that both *Pantoea* and *Bacillus* can efficiently express esterase [31,32], which participate in ester synthesis and enhances the flavor-producing function of *Daqu*, suggesting that these microorganisms may be the core drivers underlying its superior esterifying power. The high acidity characteristic of high-temperature *Daqu* (0.82–3.36 mmol/10 g), especially the peak acidity observed in HBJZ, was closely correlated with the high abundance of thermotolerant fungi (e.g., *Aspergillus* and *Fusarium*) in it. This is in agreement with the findings of Tang et al. [33] and Cao et al. [34], who reported a positive correlation between thermophilic fungi, such as *Aspergillus* and *Fusarium,* and the acidity of *Daqu*. The high fermenting power of GZZY (1.23 g/100 g) reflects the metabolic adaptability of thermotolerant microorganisms, including *Kroppenstedtia*, *Lentibacillus*, and *Bacillus* to high-temperature environments. These microbes are the primary agents responsible for decomposing starch and proteins as well as generating flavor precursors [35,36].

Metagenomic analysis revealed that the microbial community structure of *Daqu* is jointly shaped by temperature and geographical origin, which aligns with the core consensus of recent research on *Daqu* microorganisms. Temperature is a key environmental factor driving the assembly of *Daqu* microbial communities, while geographical conditions and technological variations contribute to the formation of distinct origin-specific characteristics. LEfSe analysis further enabled the identification of the signature microbial taxa of *Daqu* from different producing regions. Medium-temperature *Daqu* was dominated by yeasts (e.g., *Pichia kudriavzevii* and *Saccharomyces cerevisiae*), LAB (e.g., *Latilactobacillus* and *Lactiplantibacillus*), and *Enterobacteriaceae*, which are efficient at carbohydrate metabolism and fermentation [37,38]. In contrast, high-temperature *Daqu* was primarily composed of thermotolerant fungi (e.g., *Aspergillus chevalieri* and *Thermothelomyces thermophilus*) and spore-forming bacteria (e.g., *Lentibacillus daqui* and *Kroppenstedtia eburnea*). Among these taxa, *Aspergillus* and *Kroppenstedtia* are the most active components of the microbial community. *Aspergillus* directly modulates the saccharification and fermentation performance of *Daqu* by secreting key enzymes, including amylase and protease, whereas *Kroppenstedtia* is likely to play a crucial role in the synthesis of flavor precursors, although its specific functional mechanisms require further investigation [38]. Notably, *Lentibacillus daqui* can produce saccharifying enzymes, cellulases, and neutral proteases [39]. In terms of origin specificity, medium-temperature *Daqu* from Sanmenxia, Henan (HNSMX) was enriched in *Leuconostoc* and *Saccharopolyspora*, whereas medium-temperature *Daqu* from Yibin, Sichuan (SCYB) was characterized by *Weissella* and *Klebsiella*. Such divergences can potentially be attributed to the unique climatic conditions, raw materials, and traditional production techniques associated with each region [40]. The microbial profile of *Daqu* is profoundly shaped by the climatic conditions of the production regions. The sampling locations span three distinct climate zones: (i) subtropical humid monsoon climate (Sichuan and Guizhou), characterized by mild and humid conditions throughout the year with high environmental humidity, which favors the proliferation of molds and yeasts [41]; (ii) temperate monsoon climate (Jiangsu and Shandong), featuring pronounced seasonal temperature variations and relatively low annual air humidity, which selects for stress-tolerant microbial communities with broader temperature adaptability [42]; and (iii) warm temperate semi-arid climate (Henan), with limited precipitation, large diurnal temperature fluctuations, and low humidity, which enriches xerophilic bacterial taxa while constraining fungal diversity [27]. The differences in environmental temperature, humidity, and precipitation across climate zones exert directional selective pressures on indigenous microorganisms, thereby forging the “origin fingerprint” of the *Daqu* microbial community [9,43].

Redundancy analysis (RDA) and Spearman correlation analysis revealed complex associations within the *Daqu* microecosystem. *Bacillus* was closely correlated with esterifying power, and this functional correspondence aligns with previous findings showing that *Bacillus* participates in ester synthesis by secreting esterase [44]. *Weissella* was involved in saccharification and liquefaction, which is consistent with the results of Liu et al. [45]. *Enterobacter* was identified as a key functional genus in liquefaction, corroborating the conclusion of Liu et al. that *Enterobacter* plays important structural and functional roles in artificially prepared *Daqu* [46]. Microbial interactions were characterized by synergy as the dominant mode and antagonism as the supplementary mode. The positively correlated consortium formed by LAB, yeasts, and acetic acid bacteria can promote glycolysis to produce esters and alcohols via synergistic metabolism [47]. Positive correlations among filamentous fungi ensure the stability of basic metabolic processes, such as starch degradation and acid production under high-temperature conditions. For example, Liu et al. demonstrated that the synergistic interaction between *Aspergillus* and *Talaromyces* in medium- and high-temperature *Daqu* is crucial for maintaining robust liquefaction functionality [48]. “Microbe–metabolite” association analysis indicated that *Leuconostoc* correlated with the largest number of metabolites (13 species), mostly fatty acid methyl esters, suggesting that this genus is a core contributor to the synthesis of ester flavor compounds in *Daqu*. LAB, including *Weissella* (eight species) and *Lactococcus* (seven species), were also closely associated with various flavor metabolites, further confirming the important role of LAB in *Daqu* flavor formation [49]. Overall, five fungal genera, including *Aspergillus* and *Colletotrichum,* were exclusively correlated with tetradecane, an important alkane in *Daqu*. Tetradecane may participate in microbial cell membrane construction or act as a signaling molecule to regulate community homeostasis, implying that these fungi might be the main producers or degraders of tetradecane.

Annotation results from the KEGG and CAZy databases revealed discrepancies in the metabolic functions of *Daqu*. At the KEGG Level 1 pathway classification, genes associated with metabolism accounted for over 70% of the total annotated genes in all samples, indicating that metabolism represents the core function of the *Daqu* microbial community [50,51]. At the Level 2 pathway classification, carbohydrate metabolism and amino acid metabolism were identified as the dominant functional categories. The gene abundance of carbohydrate metabolism increased with increasing temperature, with pathways, including starch, sucrose, and galactose metabolism, being significantly enriched in high-temperature *Daqu* [23,51]. In contrast, the gene abundance of amino acid metabolism decreased with increasing temperature, and pathways such as valine–leucine–isoleucine degradation and tryptophan metabolism were enriched in medium-temperature *Daqu* [52]. Under high-temperature conditions, microorganisms need to rapidly decompose carbohydrates to obtain energy to cope with heat stress [51]. On the other hand, a medium-temperature environment is more conducive to protease activity, enabling microorganisms to degrade proteins for amino acid utilization as nitrogen sources while simultaneously converting these amino acids into flavor compounds such as aldehydes and ketones [50,52]. Analysis based on the CAZy database showed that the gene abundances of glycoside hydrolases (GHs) and glycosyltransferases (GTs) in medium-temperature *Daqu* were higher than those in high-temperature *Daqu* [50,53]. The GH family includes enzymes related to starch liquefaction and saccharification, and their high abundance constitutes the molecular basis for the excellent saccharifying power of medium-temperature *Daqu* [53]. In contrast, the relatively low GH/GT abundances in high-temperature *Daqu* resulted in an insufficient substrate degradation capacity. This finding also explains the technological rationale behind the practical Baijiu brewing process, in which the dosage of high-temperature *Daqu* (50%) is much higher than that of medium-temperature *Daqu* (20–25%) [29].

The metabolite categories identified in *Daqu* samples included esters, alkanes, alcohols, aldehydes and ketones, and organic acids, among which esters exhibited the highest relative abundance [50,54]. PCA revealed pronounced variations in metabolite profiles among different *Daqu* samples, and these variations were closely correlated with microbial community structures. Screening of differential metabolites further verified the interactions between microorganisms and metabolites [50,51]. A clear differentiation in metabolite composition was observed between medium- and high-temperature *Daqu*. The upregulated metabolites in medium-temperature *Daqu* were predominantly esters and fatty acids, which was consistent with the flavor compound synthesis characteristics of medium-temperature *Daqu* [54]. In contrast, the downregulated metabolites in high-temperature *Daqu* were mostly alcohols and ketones, corresponding to the products of carbohydrate metabolism. These discrepancies directly reflect the regulatory effect of *Daqu*-making temperature on the metabolic functions of microorganisms [23,51].

There were certain variations in the abundances of enzymes related to substrate degradation and characteristic flavor synthesis, and the core cause could be attributed to the synergistic effects of the *Daqu*-making environment and microbial community structure [23,51]. SCDY exhibited higher abundances of enzymes associated with liquefying power, saccharifying power, fermenting power, and synthesis of most characteristic flavor compounds (higher alcohols, tetramethylpyrazine, and phenylethanol), indicating that this sample had established an optimal microecological balance. This balance not only ensured the synergistic proliferation of fungi and bacteria involved in substrate degradation, but also provided a suitable metabolic environment for microorganisms responsible for characteristic flavor synthesis. GZZY and SDZB-M showed the second-highest abundance of enzymes related to substrate degradation and partial flavor synthesis, which might be associated with a slight deviation in temperature from the optimal range during *Daqu* production [51], resulting in mild inhibition of colonization and enzyme synthesis of some functional microorganisms [53]. In contrast, although SDZB-H had relatively high abundances of enzymes related to esterification and fatty acid synthesis, the abundances of enzymes associated with liquefying power and saccharifying power were low. It is inferred that the excessively high temperature during the production of this sample inhibited the activity of certain saccharifying fungi while enhancing the fatty acid synthesis and esterification functions of thermotolerant bacteria [54]. HBJZ also had high abundances of enzymes related to esterification and fatty acid synthesis, providing further evidence that microbial metabolic functions vary under different environmental conditions [32]. Carbohydrate catabolism represents the core functional module supporting *Daqu* fermentation and flavor formation. The efficient degradation of macromolecular substances and sustained carbon supply observed in this study reflect the high metabolic activity and functional synergy of microbial communities during *Daqu* fermentation. Microorganisms rely on the complete glycolytic and downstream metabolic networks to accomplish carbon source conversion, laying the material foundation for the synthesis of various flavor precursors [55,56]. The concurrent synthesis of esters, pyrazines, and higher alcohols collectively drives the accumulation of multiple key flavor compounds, conferring upon *Daqu* a rich and hierarchically structured flavor profile [57].

## 5. Conclusions

This study systematically investigated the microbial community structure, metabolic functions, and metabolite profiles of nine *Daqu* samples from six major Baijiu-producing regions. The results demonstrate that temperature is the primary driver shaping microbial community assembly and functional differentiation in *Daqu*. Medium-temperature *Daqu* exhibits higher microbial diversity and saccharifying activity, with yeasts and bacteria co-dominating fermentation, while high-temperature *Daqu* shows enhanced carbohydrate degradation capacity associated with thermotolerant microbiota. LEfSe analysis identified stable microbial biomarkers distinguishing the two *Daqu* types, and metabolomic profiling revealed distinct metabolite signatures—medium-temperature *Daqu* is enriched in esters and fatty acids, whereas high-temperature *Daqu* contains higher proportions of alcohols and ketones. Microbial–metabolite network analysis further revealed a functionally cooperative consortium dominated by LAB, yeasts, and acetic acid bacteria that ensures fermentation robustness through coordinated metabolism. These findings establish a temperature-driven framework for microbial functional differentiation and flavor development in *Daqu*, providing a scientific basis for quality-oriented regulation and process optimization in Baijiu production.

## Data Availability

The original contributions presented in the study are included in the article, further inquiries can be directed to the corresponding authors.
